# The efficacy of duloxetine: A comprehensive summary of results from MMRM and LOCF_ANCOVA in eight clinical trials

**DOI:** 10.1186/1471-244X-4-26

**Published:** 2004-09-08

**Authors:** Craig H Mallinckrodt, Joel Raskin, Madelaine M Wohlreich, John G Watkin, Michael J Detke

**Affiliations:** 1 Eli Lilly & Company, Lilly Corporate Center, Indianapolis, IN, USA; 2 Departments of Psychiatry, Harvard Medical School, Boston MA; McLean Hospital, Belmont MA; and Indiana University School of Medicine, Indianapolis, IN, USA

## Abstract

**Background:**

A mixed-effects model repeated measures approach (MMRM) was specified as the primary analysis in the Phase III clinical trials of duloxetine for the treatment of major depressive disorder (MDD). Analysis of covariance using the last observation carried forward approach to impute missing values (LOCF_ANCOVA) was specified as a secondary analysis. Previous research has shown that MMRM and LOCF_ANCOVA yield identical endpoint results when no data are missing, while MMRM is more robust to biases from missing data and thereby provides superior control of Type I and Type II error compared with LOCF_ANCOVA. We compared results from MMRM and LOCF_ANCOVA analyses across eight clinical trials of duloxetine in order to investigate how the choice of primary analysis may influence interpretations of efficacy.

**Methods:**

Results were obtained from the eight acute-phase clinical trials that formed the basis of duloxetine's New Drug Application for the treatment of MDD. All 202 mean change analyses from the 20 rating scale total scores and subscales specified *a priori *in the various protocols were included in the comparisons.

**Results:**

In 166/202 comparisons (82.2%), MMRM and LOCF_ANCOVA agreed with regard to the statistical significance of the differences between duloxetine and placebo. In 25/202 cases (12.4%), MMRM yielded a significant difference when LOCF_ANCOVA did not, while in 11/202 cases (5.4%), LOCF_ANCOVA produced a significant difference when MMRM did not. In 110/202 comparisons (54.4%) the p-value from MMRM was lower than that from LOCF_ANCOVA, while in 69/202 comparisons (34.2%), the p-value from LOCF_ANCOVA was lower than that from MMRM. In the remaining 23 comparisons (11.4%), the p-values from LOCF_ANCOVA and MMRM were equal when rounded to the 3^rd ^decimal place (usually as a result of both p-values being < .001). For the HAMD_17 _total score, the primary outcome in all studies, MMRM yielded 9/12 (75%) significant contrasts, compared with 6/12 (50%) for LOCF_ANCOVA. The expected success rate was 80%.

**Conclusions:**

Important differences exist between MMRM and LOCF_ANCOVA. Empirical research has clearly demonstrated the theoretical advantages of MMRM over LOCF_ANCOVA. However, interpretations regarding the efficacy of duloxetine in MDD were unaffected by the choice of analytical technique.

## Background

Treatment effects are often evaluated by comparing change over time in outcome measures. However, valid analyses of longitudinal data can be problematic, particularly if some data are missing for reasons related to the outcome measure [1,2]. Since the problem of missing data is almost ever-present in clinical trials, numerous methods for handling missingness have been proposed, examined, and implemented [3].

A common method of analyzing clinical trial data is to use analysis of variance or analysis of covariance (ANOVA or ANCOVA) with missing data imputed by the last observation carried forward approach (LOCF_ANCOVA). The popularity of LOCF_ANCOVA may be due to its simplicity, and also the belief that violations of the restrictive assumptions inherent to LOCF_ANCOVA lead to a conservative analysis [4]. Considerable advances in statistical methodology, and in our ability to implement these methods, have been made in recent years. Thus, methods that require less restrictive assumptions than LOCF_ANCOVA are now readily implemented. For example, likelihood-based repeated measures approaches have a number of theoretical and practical advantages for analysis of longitudinal data with dropout [4].

One such method, termed MMRM (Mixed Model Repeated Measures [5]), has been studied extensively in the context of neuropsychiatric clinical trials [6-9]. In these studies, MMRM was found to be more robust to biases from missing data than LOCF_ANCOVA, and thereby provided superior control of Type I and Type II errors. The LOCF_ANCOVA method was shown to underestimate treatment group differences in some scenarios, while overestimating differences in others. When no data were missing, the two methods yielded identical results.

The MMRM approach was specified as the primary analysis in the Phase III clinical trials of duloxetine for the treatment of major depressive disorder (MDD), while LOCF_ANCOVA was specified as a secondary analysis. In the present investigation, we provide a comprehensive summary of results from MMRM and LOCF_ANCOVA in the eight acute-phase clinical trials that formed the basis for duloxetine's New Drug Application (NDA) for MDD. The primary objective of this investigation was to determine whether differences in results between MMRM and LOCF_ANCOVA influenced conclusions regarding the efficacy of duloxetine.

## Methods

### Data

The data source for this investigation was the eight acute-phase clinical trials in which duloxetine was compared with placebo in the treatment of MDD. Relevant details of these studies are highlighted in Table 1.

**Table 1:** Summary of studies included in the comparisons between MMRM and LOCF_ANCOVA

**Study**	**Treatment Duration**	**Drug**	**Number of Patients**	**Drug Dose & Design**
**1**	9 weeks	Placebo	122	-
		Duloxetine	123	60 mg/d (QD)
**2**	9 weeks	Placebo	139	-
		Duloxetine	128	60 mg/d (QD)
**3**	8 weeks	Placebo	70	-
		Duloxetine	70	40–120 mg/d (20 mg-60 mg BID)
		Fluoxetine	33	20 mg/d (QD)
**4**	8 weeks	Placebo	75	-
		Duloxetine	82	40–120 mg/d (20 mg-60 mg BID)
		Fluoxetine	37	20 mg/d (QD)
**5**	8 weeks	Placebo	90	-
		Duloxetine	91	40 mg/d (20 mg BID)
		Duloxetine	84	80 mg/d (40 mg BID)
		Paroxetine	89	20 mg/d (QD)
**6**	8 weeks	Placebo	89	-
		Duloxetine	86	40 mg/d (20 mg BID)
		Duloxetine	91	80 mg/d (40 mg BID)
		Paroxetine	87	20 mg/d (QD)
**7**	8 weeks	Placebo	93	-
		Duloxetine	95	80 mg/d (40 mg BID)
		Duloxetine	93	120 mg/d (60 mg BID)
		Paroxetine	86	20 mg/d (QD)
**8**	8 weeks	Placebo	99	-
		Duloxetine	93	80 mg/d (40 mg BID)
		Duloxetine	103	120 mg/d (60 mg BID)
		Paroxetine	97	20 mg/d (QD)

Results are summarized from all rating scale total scores, subscales, and global assessments that were specified *a priori *in the various protocols to be analyzed for mean change from baseline to endpoint, and were collected at more than one postbaseline time point (Table 2). Efficacy measures that were assessed only at baseline and endpoint were not included in this summary because repeated measures analyses were not possible for these outcomes. Thus, the present investigation included every rating scale total score and subscale from every clinical trial relevant to duloxetine's NDA for an indication in major depression. In total, 20 efficacy and health outcome variables were included in the summary of MMRM and LOCF_ANCOVA. Some of the eight trials included multiple dose arms; therefore, some outcomes were assessed in as many as 12 comparisons with placebo.

**Table 2:** Outcomes included in the summary of results.

17-item Hamilton Rating Scale for Depression (HAMD_17_)
Total score [24]
Core subscale (items 1, 2, 3, 7, 8) (not published)
Maier subscale (items 1, 2, 7, 8, 9, 10) [25]
Anxiety/Somatization subscale (items 10, 11, 12, 13, 15, 17) [26]
Retardation subscale (items 1, 7, 8, 14) [27]
Sleep subscale (items 4, 5, 6) [27]
Montgomery-Asberg Depression Rating scale [28]
Hamilton Anxiety Rating Scale total score [29]
Clinical Global Impression of Severity [26]
Patient Global Impression of Improvement [26]
Visual Analog Scale for pain [30]
Overall pain severity
Headaches
Back pain
Shoulder pain
Time in pain while awake
Interference with daily activities
Somatic Symptom Inventory [31]
26 Item total score
28 Item total score (includes 2 additional questions on painful physical symptoms)
Quality of Life in Depression Scale total score [32]

Comparisons of MMRM and LOCF_ANCOVA focused on contrasts between duloxetine and placebo. However, six of the studies also included known effective antidepressants approved for marketing in the United States and other countries. Contrasts between duloxetine and the active comparators are not included in this summary since these results may draw attention to the drug versus drug results and detract from the primary focus of comparing MMRM with LOCF_ANCOVA.

### Statistical analysis

This summary makes no attempt to provide formal statistical comparisons of results from MMRM and LOCF_ANCOVA. Previous research has demonstrated conclusively that in the absence of missing data the two methods yield identical endpoint contrasts, while differences do exist in the presence of subject dropout [6-9]. Furthermore, formal statistical comparisons are typically applied to random samples obtained from larger populations in order to assess the uncertainty associated with the sampling. However, the eight studies included in this summary are not a sample, but rather represent all of the acute-phase, double-blind, placebo-controlled trials of duloxetine. Thus, there is no uncertainty associated with sampling. Consequently, results from the two methods need only be summarized in order to assess how differences between the methods may influence overall conclusions regarding the efficacy of duloxetine.

Three overall summary measures were used to compare results from the two analytic techniques: 1) With regard to statistical significance of the difference between duloxetine and placebo, the proportion of outcomes showing agreement between MMRM and LOCF_ANCOVA was compared with the proportion of outcomes for which MMRM and LOCF_ANCOVA yielded disparate results; 2) The proportion of outcomes for which MMRM yielded the lowest p-value was compared with the corresponding proportion for LOCF_ANCOVA; 3) The number of outcomes for which "substantial evidence of efficacy" was demonstrated. In regulatory settings, the criterion for substantial evidence of efficacy is frequently the demonstration of a statistically significant advantage over placebo in two or more studies. This criterion was utilized here to define substantial evidence of efficacy for a particular outcome.

The frequency of lower p-values provides a "fine-tuned" measure of sensitivity of the two analytic methods. However, in certain cases such an assessment may actually be misleading. For example, to distinguish between p= .800 and p = .810, or between p =. 002 and p =. 003, implies that the methods yielded different results when in fact the similarities far outweigh the differences. Hence, it is equally appropriate to simply categorize based upon the presence or absence of a significant difference. Furthermore, given the large number of outcomes assessed across the eight studies, it would not be surprising to see the two methods disagree with regard to statistical significance on at least a small number of outcomes. Therefore, perhaps the most clinically meaningful summary measure is the number of outcomes for which substantial evidence of efficacy was demonstrated.

Three outcomes were selected for more detailed presentation of results: the 17-item Hamilton Rating Scale for Depression (HAMD_17_) total score, the HAMD_17 _Maier subscale, and the Visual Analog Scale (VAS) for overall pain severity. The HAMD_17 _total score was an obvious choice as it was the primary outcome in all studies. The other outcomes were selected since they are frequently focal points in manuscripts and presentations regarding duloxetine's efficacy. Finally, we provide case studies to help explain how and why results from MMRM and LOCF_ANCOVA may differ.

MMRM and LOCF_ANCOVA analyses were specified in the Phase III duloxetine protocols as follows. In LOCF_ANCOVA analyses, change from baseline to the last observation was the dependent variable. Treatment and investigative site were included as categorical independent variables, and baseline severity was included as a covariate. In MMRM analyses, change from baseline to all postbaseline times was the dependent variable. Independent variables included the fixed, categorical effects of investigative site, treatment, time, and the treatment-by-time interaction, along with the continuous covariates of baseline severity and the baseline severity by time interaction. Parameters were estimated using Restricted Maximum Likelihood with the Newton-Raphson algorithm. The protocols specified an algorithm for choosing the best fitting covariance structure. In all cases an unstructured matrix provided the best fit. Hence, within-patient errors were modeled using an unstructured covariance matrix.

## Results

The protocols for the eight studies in the duloxetine NDA specified *a priori *a total of 202 mean change analyses for the 20 rating scale total scores or subscales. The frequency of significant outcomes and the frequency of higher/lower p-values for each analytic technique are summarized in Table 3. MMRM and LOCF_ANCOVA agreed with regard to substantial evidence of efficacy for 18 of the 20 outcomes, with each analysis yielding substantial evidence for 15 outcomes. That is, MMRM and LOCF_ANCOVA both found substantial evidence of efficacy for 14 outcomes; both methods did not find substantial evidence for four outcomes; and each method found substantial evidence when the other did not for one outcome (Table 3).

**Table 3:** Summary of results from MMRM and LOCF_ANCOVA in duloxetine clinical trials.

**Outcome**	**Frequency of significant outcomes**^1^**MMRM LOCF_ANCOVA**		**P-value lower with MMRM LOCF_ANCOVA**		**Outcome significant with MMRM but not LOCF_ANCOVA**	**Outcome significant with LOCF_ANCOVA but not MMRM**
**HAMD**_17_						
Total score	9\12	6\12	9\12	1\12	3\12	0\12
Core subscale	9\12	8\12	5\12	3\12	2\12	1\12
Maier subscale	10\12	9\12	6\12	2\12	2\12	1\12
Anxiety subscale	6\12	5\12	7\12	3\12	2\12	1\12
Retardation subscale	8\12	6\12	6\12	3\12	4\12	2\12
Sleep subscale	1\12	1\12	10\12	2\12	0\12	0\12
**MADRS Total score**	5\10	3\10	7\10	2\10	2\10	0\10
**HAMA Total score**	3\10	3\10	4\10	5\10	0\10	0\10
**CGI-Severity**	6\12	6\12	8\12	2\12	0\12	0\12
**CGI-Improvement**	1\2	1\2	2\2	0\2	0\2	0\2
**PGI-Improvement**	9\12	7\12	8\12	2\12	2\12	0\12
**Somatic Symptom Inventory**						
26-item Average score	2\10	2\10	7\10	3\10	0\10	0\10
28-item Average score	3\10	2\10	7\10	3\10	1\10	0\10
**VAS Pain Severity**						
Overall pain	3\10	4\10	2\10	8\10	1\10	2\10
Headaches	1\10	0\10	5\10	5\10	1\10	0\10
Back pain	2\10	2\10	6\10	4\10	1\10	1\10
Shoulder pain	2\10	1\10	3\10	6\10	2\10	1\10
Daily activities	1\10	0\10	2\10	8\10	1\10	0\10
Pain while awake	2\10	2\10	5\10	5\10	1\10	1\10
**QLDS Total score**	1\4	2/4	1\4	2\4	0\4	1\4
**Totals**	**84\202**	**70\202**	**110\202**	**69\202**	**25\202**	**11\202**

1. Some of the eight trials included more than one dose arm. Therefore, an individual outcome could be assessed in as many as 12 comparisons with placebo.

In 166/202 outcomes (82.2%), MMRM and LOCF_ANCOVA agreed with regard to the statistical significance of the difference between duloxetine and placebo. In 25 cases (12.4%) MMRM yielded a significant difference whereas LOCF_ANCOVA did not, while in 11 cases (5.4%) LOCF_ANCOVA yielded a significant difference when MMRM did not.

Both methods tended to yield significance more frequently in depression rating scales and subscales than in outcomes related to somatic and painful physical symptoms. The studies were generally underpowered for these secondary somatic and pain outcomes owing to the greater variance in changes score for these outcomes. For example, the variance in VAS overall pain severity was approximately nine-fold greater than the variance in HAMD_17 _total scores, leading to a three-fold greater standard error.

In 110 of the 202 outcomes (54.4%) the p-value from MMRM was lower than that from LOCF_ANCOVA, while in 69 cases (34.2%) the p-value from LOCF_ANCOVA was lower than that from MMRM. In the remaining 23 cases (11.4%) the p-values from LOCF_ANCOVA and MMRM were equal when rounded to the 3^rd ^decimal place (usually as a result of both p-values being < .001).

More detailed results from the three focus outcomes (HAMD_17 _total score, HAMD_17 _Maier subscale, and VAS overall pain) are presented in Table 4. In the case of the HAMD_17 _total score, the advantage of duloxetine over placebo in mean change from baseline to endpoint from MMRM analyses was greater than the corresponding advantage from LOCF_ANCOVA in 9/12 comparisons (Table 4). In 9/12 comparisons the p-value from MMRM was lower than that from LOCF_ANCOVA, while LOCF_ANCOVA yielded a smaller p-value in one case, and p-values were identical in the two remaining cases. In 3/12 comparisons, MMRM yielded a significant difference when LOCF_ANCOVA did not, but in no instance did LOCF_ANCOVA produce a significant difference when MMRM did not. When averaging results across all eight studies, the advantage of duloxetine over placebo in HAMD_17 _total score was 2.4 from MMRM analyses compared with 2.0 from LOCF_ANCOVA. Thus, the advantage of duloxetine over placebo, based on LOCF_ANCOVA results, was approximately 83% as large as the advantage from MMRM.

**Table 4:** MMRM and LOCF_ANCOVA analysis of three focus outcomes from duloxetine clinical trials.

	**Study 1**		**Study 2**		**Study 3**		**Study 4**		**Study 5**				**Study 6**				**Study 7**				**Study 8**			
**Dose**	60 mg QD		60 mg QD		60 mg BID		60 mg BID		20 mg BID		40 mg BID		20 mg BID		40 mg BID		40 mg BID		60 mg BID		40 mg BID		60 mg BID	
	Δ	p	Δ	p	Δ	p	Δ	p	Δ	p	Δ	p	Δ	p	Δ	p	Δ	p	Δ	p	Δ	p	Δ	p

**HAMD**_17_**Total Score**																								
**MMRM**	4.9	< .001	2.2	.024	3.1	.009	0.9	.415	1.4	.143	1.5	.116	2.4	.034	3.6	.002	2.2	.001	3.3	< .001	1.4	.045	1.6	.014
**LOCF**	3.8	< .001	1.7	.048	2.1	.066	0.4	.681	1.2	.222	1.5	.138	2.4	.022	3.1	.003	2.2	.007	3.0	< .001	0.9	.253	1.5	.054
**HAMD**_17 _**Maier Subscale**																								
**MMRM**	2.8	< .001	1.6	.003	2.0	.005	0.7	.282	1.2	.037	1.4	.012	1.1	.068	1.7	.005	1.4	< .001	2.0	< .001	0.9	.022	1.2	.002
**LOCF**	2.3	< .001	1.4	.007	1.0	.030	0.6	.481	1.0	.058	1.3	.012	1.5	.028	1.8	.004	1.4	.001	1.9	< .001	0.7	.090	1.0	.014
**VAS Overall Pain Severity**																								
**MMRM**	5.9	.055	4.4	.135	NA		NA		0.3	.931	1.2	.731	1.0	.771	7.4	.035	5.5	.085	6.3	.050	5.6	.044	1.3	.625
**LOCF**	6.9	.019	5.2	.037	NA		NA		3.5	.573	3.5	.647	3.2	.710	7.1	.048	6.1	.063	5.6	.086	7.8	.014	5.5	.066

NA = not assessed

Similar results were obtained for the Maier subscale. Thus, the advantage of duloxetine over placebo in mean change from baseline to endpoint from MMRM was greater than that from LOCF_ANCOVA in 9/12 comparisons (Table 4). The p-value from MMRM was lower than that from LOCF_ANCOVA in 6/12 comparisons, while LOCF_ANCOVA produced a lower p-value in 2 cases, and p-values were identical in the remaining four cases. In 2/12 comparisons MMRM yielded a significant difference when LOCF_ANCOVA did not, while there was one instance in which LOCF_ANCOVA yielded a significant difference when MMRM did not. Averaged over all eight studies, the advantage of duloxetine over placebo in mean Maier subscale score was 1.5 from MMRM analyses compared with 1.3 from LOCF_ANCOVA. Thus, the advantage of duloxetine over placebo based on LOCF_ANCOVA results was approximately 87% as large as the advantage from MMRM.

For VAS overall pain severity, the advantage of duloxetine over placebo from MMRM analyses was greater than the corresponding advantage from LOCF_ANCOVA in 2/10 comparisons (Table 4). The p-value from MMRM was lower than that from LOCF_ANCOVA in 2/10 comparisons, while in the remaining 8 comparisons the p-value from LOCF_ANCOVA was lower than that from MMRM. In 1 comparison MMRM yielded a significant difference when LOCF_ANCOVA did not, while in 2 comparisons LOCF_ANCOVA yielded a significant difference when MMRM did not. Over all eight studies, the average advantage of duloxetine over placebo in VAS overall pain severity was 3.9 from MMRM analyses compared with 5.4 from LOCF_ANCOVA. Thus, the advantage of duloxetine based on LOCF_ANCOVA results was approximately 138% as large as the advantage from MMRM.

### Case Studies

Mean changes in HAMD_17 _total score and VAS overall pain severity from two studies (Studies 1 and 2) are used to further illustrate MMRM and LOCF_ANCOVA (analysis of variance with missing data imputed via last observation carried forward) results. Results from these studies were originally reported by Detke et al [10,11]. In both studies the advantage of duloxetine over placebo in HAMD_17 _total score tended to increase over time whereas duloxetine's advantage in VAS overall pain was greatest at intermediate visits (Tables 5 and 6).

**Table 5:** MMRM and LOCF_ANCOVA analyses of HAMD_17_ total score in Studies 1 and 2.

		**STUDY 1**				**STUDY 2**			
**MMRM**									
**THERAPY**	**Week**	**N**	**Mean change**	**Std.error**	**p-value**	**N**	**Mean change**	**Std.error**	**p-value**
DULOX	1	121	-2.89	0.36	.435	123	-2.89	0.38	.601
PLACEBO	1	115	-2.50	0.37		136	-2.64	0.36	
DULOX	2	112	-5.72	0.49	< .001	109	-5.54	0.48	.071
PLACEBO	2	110	-3.39	0.50		129	-4.43	0.45	
DULOX	3	105	-7.37	0.53	< .001	108	-6.82	0.55	.287
PLACEBO	3	103	-4.58	0.54		122	-6.06	0.52	
DULOX	5	100	-8.76	0.60	< .001	98	-8.58	0.66	.116
PLACEBO	5	101	-5.74	0.60		111	-7.20	0.62	
DULOX	7	91	-9.93	0.64	< .001	89	-10.14	0.69	.008
PLACEBO	7	93	-5.82	0.65		97	-7.69	0.65	
DULOX	9	84	-10.91	0.70	< .001	81	-10.46	0.71	.024
PLACEBO	9	89	-6.05	0.69		90	-8.29	0.67	
**LOCF_ANCOVA**									
DULOX	LOCF_ANCOVA	121	-9.47	0.63	< .001	123	-8.75	0.71	.048
PLACEBO	LOCF_ANCOVA	115	-5.67	0.66		136	-7.02	0.68	

**Table 6:** MMRM and LOCF_ANCOVA analyses of VAS overall pain severity in Studies 1 and 2.

		**STUDY 1**				**STUDY 2**			
**MMRM**									
**THERAPY**	**Week**	**N**	**Mean change**	**Std.error**	**p-value**	**N**	**Mean change**	**Std.error**	**p-value**
DULOX	1	120	-2.69	1.97	.181	121	-2.02	1.89	.157
PLACEBO	1	113	1.04	2.04		134	1.38	1.79	
DULOX	2	113	-8.20	1.87	.003	108	-6.95	2.04	.003
PLACEBO	2	108	-0.42	1.93		127	0.91	1.90	
DULOX	3	105	-10.46	1.95	.005	106	-9.81	1.90	< .001
PLACEBO	3	101	-2.56	2.01		119	-1.59	1.78	
DULOX	5	99	-8.68	2.20	.028	95	-9.70	2.05	.011
PLACEBO	5	99	-1.85	2.24		108	-2.92	1.92	
DULOX	7	91	-10.25	2.34	.016	87	-8.91	2.12	.254
PLACEBO	7	92	-2.26	2.38		94	-5.76	2.01	
DULOX	9	83	-8.68	2.18	.055	80	-10.05	2.24	.135
PLACEBO	9	88	-2.80	2.20		88	-5.65	2.12	
**LOCF_ANCOVA**									
DULOX	LOCF_ANCOVA	121	-8.31	2.09	.019	121	-10.39	2.05	.037
PLACEBO	LOCF_ANCOVA	114	-1.40	2.18		134	-5.22	1.94	

In the case of the HAMD_17 _total score, advantages for duloxetine over placebo at endpoint (Week 9) from MMRM in Studies 1 and 2 were 4.86 (p < .001) and 2.17 (p = .024), respectively. The corresponding advantages from LOCF_ANCOVA were 3.80 (p < .001) and 1.73 (p = .048). Although the differences were significant for both methods in both studies, MMRM yielded treatment contrasts that were approximately 25% greater than LOCF_ANCOVA.

For VAS overall pain, the advantage of duloxetine over placebo at endpoint from MMRM in Studies 1 and 2 were 5.88 (p = .055) and 4.40 (p = .135), respectively. The corresponding advantages from LOCF_ANCOVA were 6.91 (p = .019) and 5.17 (p = .037). In both studies, the endpoint differences were significant from LOCF_ANCOVA, but not from MMRM. The LOCF_ANCOVA treatment contrasts were approximately 15% greater than those from MMRM.

Standard errors from LOCF_ANCOVA were approximately 5% smaller than the Week 9 standard errors from MMRM for both the HAMD_17 _total score and VAS overall pain.

## Discussion

In many areas of clinical research, the impact of missing data can be profound [2,12-14]. Traditional approaches to analyses of data from clinical trials with dropouts, such as LOCF_ANCOVA, have focused on ease of implementation and interpretation. However, simple methods rely upon assumptions that are often unrealistic. For example, LOCF_ANCOVA assumes that patient dropout is completely random, i.e. it is unrelated to the outcome being analyzed. Hence, in an analysis of efficacy data, LOCF_ANCOVA assumes that patients do not drop out due to lack of efficacy. The LOCF_ANCOVA approach also assumes that, for those patients who drop out, their observations would not have changed had they stayed in the trial. When these assumptions do not hold true, estimates of treatment effects and associated standard errors may be biased [2-4,6,7,15-17].

Considerable advances in statistical methodology, and in our ability to implement these methods, have been made in recent years. Methods such as MMRM, which require less restrictive assumptions regarding missing data, may now be easily implemented with standard software [4,5,18,19].

No universally superior approach to analysis of longitudinal data exists. However, a series of studies [6-9] demonstrated empirically what may have been anticipated from statistical theory – namely that the MMRM approach, while providing no guarantee of immunity from bias due to subject dropout, was a sensible analytic choice for many clinical trial scenarios. MMRM has repeatedly been shown to provide adequate control of Type I (false positive) and Type II (false negative) errors in a wide variety of situations modeled after neuropsychiatric clinical trials. In these head-to-head comparisons involving 456,000 data sets, the LOCF_ANCOVA approach did not perform as well as MMRM. We therefore specified MMRM as the primary analysis and LOCF_ANCOVA as a secondary analysis in the Phase III clinical trials of duloxetine in the treatment of MDD.

Similar results regarding control of Type I and Type II error for LOCF_ANCOVA and mixed-effects model analyses have been obtained independently [16,20-22]. Furthermore, following an independent investigation of data from two placebo- and active-comparator controlled duloxetine trials, in which treatments were coded A, B, C, *etc. *to blind analysts to the treatment names, Molenberghs et al [4] concluded that MMRM analysis was a sensible choice for those data.

The theoretical differences between MMRM and LOCF_ANCOVA have been summarized [5,18], established empirically [6-9], and proven mathematically [4]. However, we are unaware of any previous investigations of how these differences manifest themselves in efficacy assessments of a new medicinal product.

The VAS pain results highlight a limitation of endpoint analyses of any type, namely that they provide only a snapshot view of the response profile. From LOCF_ANCOVA analysis, one can only conclude that drug was superior to placebo at endpoint. However, MMRM analysis reveals that drug had a significant effect early in the trials, but that advantage was somewhat transitory as the placebo group tended to "catch up" over time. In order to understand the response profile of a drug, the entire longitudinal profile should be considered [2]. From MMRM, the entire profile can be assessed from the same analysis that provided the primary result (the contrast at endpoint).

In the duloxetine database, results from MMRM and LOCF_ANCOVA were in general agreement regarding substantial evidence of efficacy and frequency of significant differences. However, MMRM tended to be more sensitive to drug-placebo differences for outcomes related to overall depressive symptoms and core emotional symptoms of depression, with mean advantages over placebo that were 10% to 20% greater than LOCF_ANCOVA. However, MMRM did not universally increase duloxetine's advantage over placebo in comparison to results from LOCF_ANCOVA. For example, in somatic and painful physical symptom outcomes, results from LOCF_ANCOVA showed mean advantages over placebo that were approximately 40% greater than that from MMRM.

Therefore, while the overall conclusions regarding the efficacy of duloxetine were unaffected by the choice of analytic method, this should not mask the important differences between MMRM and LOCF_ANCOVA. The advantages of MMRM and similar methods over LOCF_ANCOVA have been conclusively demonstrated in many studies and are evident in the duloxetine data.

Khan et al [23] compiled a database from FDA summaries of efficacy for all antidepressants approved between 1985 and 2000. Less than half of the studies – which were analyzed using LOCF_ANCOVA as the primary analysis – found significant advantages for drug over placebo. These studies were generally anticipated to have at least 80% power and, if the analysis worked as expected, the success rate would be 80%. Therefore, LOCF_ANCOVA was less sensitive to the drug effects than anticipated. In the duloxetine database, MMRM yielded a 75% success rate for the primary outcome measure (HAMD_17 _total score), while LOCF_ANCOVA produced a 50% success rate, in comparison to the expected rate of 80%. While many factors may reduce the success rate in Phase III clinical trials, the use of a statistical method with known inflation of Type II error (false negative results) is an obvious suspect.

An unduly high rate of false negative results could be especially problematic in early phases of drug development where only one or two chances exist to make the correct decision regarding the efficacy of a drug. It is noteworthy that one of the instances when MMMRM yielded a significant difference on the primary outcome (HAMD_17 _total score) when LOCF did not was in the Phase II study (Study 3).

Also consider that across all therapeutic areas only about 50% of the molecules that enter Phase III testing receive regulatory approval. Many factors may reduce the success rate of Phase III development. However, the use in Phase II of a statistical method with known inflation of Type I error (false positive results) is an obvious suspect. Thus, the unexpectedly low success rate in Phase III is consistent with the conclusion that LOCF_ANCOVA inflates Type I error (as a result of an unduly high rate of false positive results in Phase II).

Hence, results from the duloxetine NDA are consistent with research suggesting a move away from LOCF_ANCOVA and other simple analytic techniques to methods such as MMRM that are more robust to the biases from missing data.

## Conclusion

Important differences exist between MMRM and LOCF_ANCOVA. Research has clearly demonstrated the advantages of MMRM over LOCF_ANCOVA. However, interpretations regarding the efficacy of duloxetine in MDD were unaffected by the choice of analytical technique.

## Competing Interests

Drs. Mallinckrodt, Raskin, Wohlreich, Watkin, and Detke are employees of Eli Lilly and Company.

## Author's Contributions

CHM designed the statistical analyses, and participated in interpretation of data and drafting of the manuscript. JR, MMW, JGW, and MJD participated in interpretation of data and drafting of the manuscript. All authors read and approved the final manuscript.

## Pre-publication history

The pre-publication history for this paper can be accessed here:


